# Bis(5-amino-3-carb­oxy-1*H*-1,2,4-triazol-4-ium) sulfate dihydrate

**DOI:** 10.1107/S1600536811013882

**Published:** 2011-04-16

**Authors:** Amira Ouakkaf, Fadila Berrah, Sofiane Bouacida, Thierry Roisnel

**Affiliations:** a Laboratoire de Chimie Appliquée et Technologie des Matériaux LCATM, Université Larbi Ben M’Hidi, 04000 Oum El Bouaghi, Algeria; b Unité de Recherche de Chimie de l’Environnement et Moléculaire Structurale, CHEMS, Faculté des Sciences Exactes, Université Mentouri Constantine 25000, Algeria; c Centre de Difractométrie X, UMR 6226 CNRS Unité Sciences Chimiques de Rennes, Université de Rennes I, 263 Avenue du Général Leclerc, 35042 Rennes, France

## Abstract

The crystal structure of the title compound, 2C_3_H_5_N_4_O_2_^+^·SO_4_^2−^·2H_2_O, displays a three-dimensional framework in which mixed infinite chains [oriented parallel to (510) and (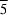
10)] inter­fere, forming tunnels extending parallel to the *c* axis. Inter­molecular O—H⋯O, N—H⋯O and O—H⋯N hydrogen bonds ensure the unity of the structure and generate centrosymmetric *R*_4_^4^(14) and *R*_4_^2^(8) rings. The S atom lies on a twofold axis.

## Related literature

For uses of 1,2,4 triazole derivatives, see: Beckmann & Brooker, (2003 ▶); Bhargava *et al.* (1981 ▶); Fujigaya *et al.* (2003 ▶); Hirota *et al.* (1991 ▶); Li *et al.* (2004 ▶); Matulková *et al.* (2008 ▶). For related hybrid compounds, see: Berrah *et al.* (2011*a* ▶,*b* ▶,*c* ▶). For hydrogen-bond motifs, see: Bernstein *et al.* (1995 ▶); Etter *et al.* (1990 ▶).
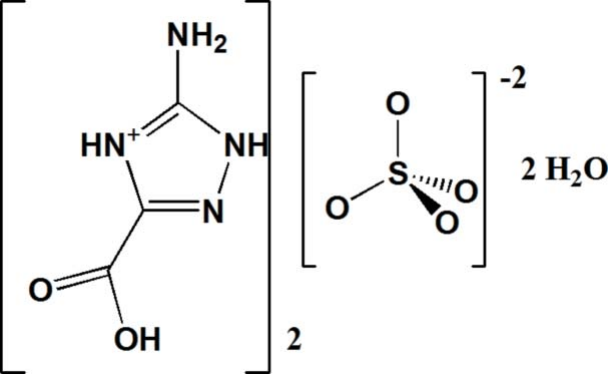


## Experimental

### 

#### Crystal data

2C_3_H_5_N_4_O_2_^+^·SO_4_^2−^·2H_2_O
*M* *_r_* = 390.31
Monoclinic, 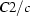

*a* = 19.4350 (9) Å
*b* = 5.8467 (2) Å
*c* = 13.3343 (6) Å
β = 109.981 (2)°
*V* = 1423.98 (10) Å^3^
*Z* = 4
Mo *K*α radiation
μ = 0.31 mm^−1^
*T* = 150 K
0.48 × 0.08 × 0.06 mm

#### Data collection

Bruker APEXII diffractometer
11018 measured reflections
1620 independent reflections
1470 reflections with *I* > 2σ(*I*)
*R*_int_ = 0.032

#### Refinement

*R*[*F* ^2^ > 2σ(*F* ^2^)] = 0.029
*wR*(*F* ^2^) = 0.077
*S* = 1.08
1620 reflections
121 parameters
H atoms treated by a mixture of independent and constrained refinement
Δρ_max_ = 0.37 e Å^−3^
Δρ_min_ = −0.41 e Å^−3^

### 

Data collection: *APEX2* (Bruker, 2001 ▶); cell refinement: *SAINT* (Bruker, 2001 ▶); data reduction: *SAINT*; program(s) used to solve structure: *SIR2002* (Burla *et al.*, 2005 ▶); program(s) used to refine structure: *SHELXL97* (Sheldrick, 2008 ▶); molecular graphics: *ORTEP-3 for Windows* (Farrugia, 1997 ▶) and *DIAMOND* (Brandenburg & Berndt, 2001 ▶); software used to prepare material for publication: *WinGX* (Farrugia, 1999 ▶).

## Supplementary Material

Crystal structure: contains datablocks global, I. DOI: 10.1107/S1600536811013882/bq2295sup1.cif

Structure factors: contains datablocks I. DOI: 10.1107/S1600536811013882/bq2295Isup2.hkl

Additional supplementary materials:  crystallographic information; 3D view; checkCIF report

## Figures and Tables

**Table 1:** Hydrogen-bond geometry (Å, °)

*D*—H⋯*A*	*D*—H	H⋯*A*	*D*⋯*A*	*D*—H⋯*A*
O1*W*—H2*W*⋯N2	0.83 (2)	2.16 (2)	2.9774 (16)	169 (2)
O1*W*—H1*W*⋯O1^i^	0.89 (2)	1.93 (2)	2.7941 (17)	163 (2)
O2—H2⋯O1*W*^ii^	0.82	1.74	2.5403 (16)	165
N1—H1⋯O4^iii^	0.86	1.94	2.7107 (16)	149
N3—H3⋯O4^iv^	0.86	1.79	2.6436 (15)	171
N4—H4*A*⋯O3^v^	0.86	2.17	2.8452 (17)	136
N4—H4*B*⋯O3^vi^	0.86	2.08	2.9255 (16)	168

Symmetry codes: (i) 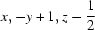; (ii) 
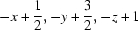
; (iii) 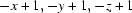; (iv) 
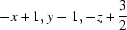
; (v) 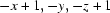; (vi) 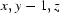.

**Fig. 1.**
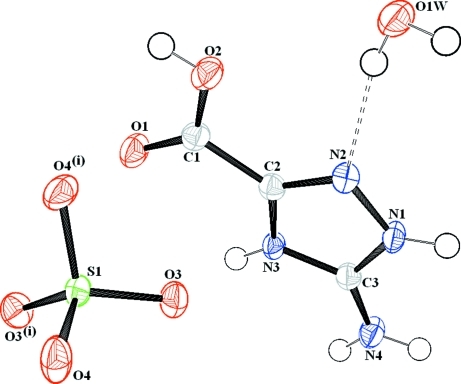
The molecular structure of the title compound with the atomic labeling scheme. Displacement are drawn at the 50% probability level. Hydrogen bonds are shown as dashed lines.

**Fig. 2.**
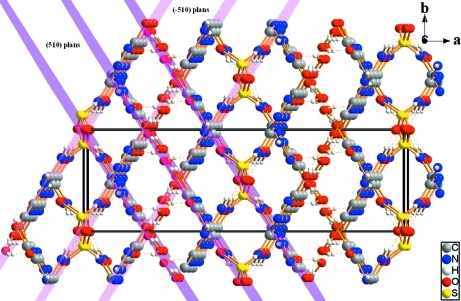
(Brandenburg & Berndt, 2001) A diagram of the three-dimensional packing of (I). (510) and (-510) planes have been presented to illustrate the mixed infinite chains. Hydrogen bonds are shown as dashed lines.

**Fig. 3.**
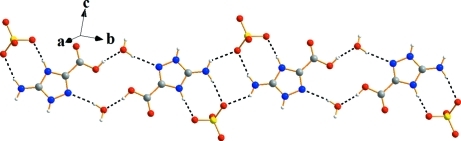
(Brandenburg & Berndt, 2001) A view of the mixed infinite chain. Hydrogen bonds are shown as dashed lines.

## supplementary crystallographic information

### Comment

1,2,4-triazole derivatives are remarkable substances exhibiting a wide range of properties; while several compounds incorporating 1,2,4-triazole moieties show pharmacological and biological activities (antidepressants, anti-inflammatory, fungicides) (Bhargava *et al.*, 1981; Hirota *et al.*, 1991; Li *et al.*, 2004), a number of them find use in material field and coordination chemistry (magnetic and non-linear optics properties, multidentate ligands) (Beckmann & Brooker, 2003; Fujigaya *et al.*, 2003; Matulková *et al.*, 2008).

Our recent investigation on some N-heterocycle compounds with inorganic acids (Berrah *et al.*, 2011*a*,*b*,*c*), has revealed their ability to generate original networks stabilized in particular by hydrogen bonds; we report herein the structure of a new hybrid compound obtained from a disubstituted 1,2,4-triazole derivative (5-amino-1,2,4 triazol-1*H*-3-carboxylic acid hydrate) and the sulfuric acid.

The molecular structure and the atom-numbering scheme of the title compound are shown in Fig. 1. The sulfur atom lies in the twofold axis and the sulfate anion shows a quite regular geometry compared with that seen in similar compounds (Berrah *et al.*, 2011*b*,*c*). The triazole ring exhibits a short distance of 1.3046 (18) Å revealing the double-bond character of the C2═N2 bond, two long distances 1.3648 (18) Å and 1.3774 (17) Å related to the single bonds C2—N3 and N1—N2, respectively. The C3—N1 and C3═ N3 bonds lengths are respectively 1.3420 (18) Å and 1.3480 (18) Å which suggests the delocalization of the double bond (N1 ≐C3 ≐N3).

The crystal structure of the title compound (Fig. 2) displays a three-dimensional framework where mixed infinite chains interfere to form tunnels parallel the *c* axis. According to their orientation, we can distinguish two kinds of chains: the first one directed along the (510) plane and the second along the (510) plane (Fig. 2). In a mixed chain, the cations adopt a head to head configuration in a way that NH_2_, of two adjacent cations, are linked (*via* N—H···O hydrogen bonds) to two sulfate anions and C—OH are linked (by O—H···O hydrogen bonds) to two water molecules (Fig. 3) (Table 1). Consequently, centro-symmetric *R*^4^_4_(14) and *R*^2^_4_(8) graph-set rings are created (Etter *et al.*, 1990; Bernstein *et al.*, 1995).

### Experimental

The title compound was synthesized by reacting 5-amino-1,2,4 triazol-1*H*-3-carboxylic acid hydrate with some excess of sulfuric acid in aqueous solution. Slow evaporation leads to well crystallized colorless needles.

### Refinement

The H atoms of the water molecules were located in difference Fourier maps and were refined with *U*_iso_(H) = 1.5*U*_eq_(O). The remaining H atoms were located in difference Fourier maps but introduced in calculated positions and treated as riding on their parent atoms (N or O) with, O—H = 0.82 Å and N—H = 0.86 Å with *U*_iso_(H) = 1.2 *U*_eq_(N) and *U*_iso_(H) = 1.5 *U*_eq_(O).

### Crystal data

**Table:**

2C_3_H_5_N_4_O_2_^+^·SO_4_^2^^−^·2H_2_O	*F*(000) = 808
*M**_r_* = 390.31	*D*_x_ = 1.821 Mg m^−^^3^
Monoclinic, *C*2/*c*	Mo *K*α radiation, λ = 0.71073 Å
*a* = 19.4350 (9) Å	Cell parameters from 1620 reflections
*b* = 5.8467 (2) Å	θ = 3.3–27.4°
*c* = 13.3343 (6) Å	µ = 0.31 mm^−^^1^
β = 109.981 (2)°	*T* = 150 K
*V* = 1423.98 (10) Å^3^	Needle, colourless
*Z* = 4	0.48 × 0.08 × 0.06 mm

### Data collection

**Table:**

Bruker APEXII diffractometer	*R*_int_ = 0.032
graphite	θ_max_ = 27.5°, θ_min_ = 3.3°
CCD rotation images, thin slices scans	*h* = −24→25
11018 measured reflections	*k* = −7→7
1620 independent reflections	*l* = −17→15
1470 reflections with *I* > 2σ(*I*)	

### Refinement

**Table:**

Refinement on *F*^2^	Primary atom site location: structure-invariant direct methods
Least-squares matrix: full	Secondary atom site location: difference Fourier map
*R*[*F*^2^ > 2σ(*F*^2^)] = 0.029	Hydrogen site location: inferred from neighbouring sites
*wR*(*F*^2^) = 0.077	H atoms treated by a mixture of independent and constrained refinement
*S* = 1.08	*w* = 1/[σ^2^(*F*_o_^2^) + (0.0343*P*)^2^ + 1.9446*P*] where *P* = (*F*_o_^2^ + 2*F*_c_^2^)/3
1620 reflections	(Δ/σ)_max_ = 0.009
121 parameters	Δρ_max_ = 0.37 e Å^−^^3^
0 restraints	Δρ_min_ = −0.41 e Å^−^^3^

### Special details

**Table:**

Geometry. All e.s.d.'s (except the e.s.d. in the dihedral angle between two l.s. planes) are estimated using the full covariance matrix. The cell e.s.d.'s are taken into account individually in the estimation of e.s.d.'s in distances, angles and torsion angles; correlations between e.s.d.'s in cell parameters are only used when they are defined by crystal symmetry. An approximate (isotropic) treatment of cell e.s.d.'s is used for estimating e.s.d.'s involving l.s. planes.
Refinement. Refinement of *F*^2^ against ALL reflections. The weighted *R*-factor *wR* and goodness of fit *S* are based on *F*^2^, conventional *R*-factors *R* are based on *F*, with *F* set to zero for negative *F*^2^. The threshold expression of *F*^2^ > σ(*F*^2^) is used only for calculating *R*-factors(gt) *etc*. and is not relevant to the choice of reflections for refinement. *R*-factors based on *F*^2^ are statistically about twice as large as those based on *F*, and *R*- factors based on ALL data will be even larger.

### Fractional atomic coordinates and isotropic or equivalent isotropic displacement parameters (Å^2^)

**Table:**

	*x*	*y*	*z*	*U*_iso_*/*U*_eq_	
C1	0.32927 (7)	0.3566 (2)	0.60560 (11)	0.0136 (3)	
C2	0.35578 (7)	0.2304 (2)	0.52873 (11)	0.0123 (3)	
C3	0.40656 (7)	−0.0381 (2)	0.46589 (11)	0.0129 (3)	
N1	0.38002 (6)	0.1265 (2)	0.39270 (9)	0.0138 (3)	
H1	0.3827	0.125	0.3296	0.017*	
N2	0.34797 (6)	0.2981 (2)	0.43222 (9)	0.0138 (3)	
N3	0.39065 (6)	0.0245 (2)	0.55278 (9)	0.0123 (2)	
H3	0.4005	−0.0502	0.6116	0.015*	
N4	0.44239 (7)	−0.2252 (2)	0.45703 (10)	0.0169 (3)	
H4A	0.451	−0.2519	0.399	0.02*	
H4B	0.4571	−0.3201	0.5093	0.02*	
O1	0.33947 (6)	0.28073 (19)	0.69430 (8)	0.0188 (2)	
O2	0.29439 (6)	0.54386 (18)	0.56322 (8)	0.0186 (2)	
H2	0.2817	0.612	0.6077	0.028*	
O3	0.48758 (5)	0.50548 (17)	0.65377 (8)	0.0158 (2)	
O4	0.56503 (6)	0.79464 (19)	0.76558 (8)	0.0190 (2)	
S1	0.5	0.64445 (8)	0.75	0.01101 (13)	
O1W	0.25419 (6)	0.69140 (19)	0.32536 (9)	0.0194 (2)	
H1W	0.2751 (11)	0.724 (4)	0.2772 (17)	0.029*	
H2W	0.2786 (11)	0.586 (4)	0.3624 (17)	0.029*	

### Atomic displacement parameters (Å^2^)

**Table:**

	*U*^11^	*U*^22^	*U*^33^	*U*^12^	*U*^13^	*U*^23^
C1	0.0118 (6)	0.0150 (7)	0.0148 (7)	−0.0021 (5)	0.0054 (5)	−0.0017 (5)
C2	0.0117 (6)	0.0128 (6)	0.0122 (7)	−0.0015 (5)	0.0037 (5)	−0.0008 (5)
C3	0.0119 (6)	0.0151 (7)	0.0117 (6)	−0.0026 (5)	0.0040 (5)	−0.0008 (5)
N1	0.0177 (6)	0.0155 (6)	0.0097 (6)	0.0011 (5)	0.0066 (5)	−0.0003 (4)
N2	0.0150 (6)	0.0143 (6)	0.0126 (6)	−0.0001 (4)	0.0053 (5)	−0.0008 (4)
N3	0.0145 (5)	0.0138 (6)	0.0093 (5)	0.0010 (4)	0.0052 (4)	0.0015 (4)
N4	0.0220 (6)	0.0162 (6)	0.0145 (6)	0.0034 (5)	0.0087 (5)	−0.0002 (5)
O1	0.0230 (5)	0.0213 (5)	0.0155 (5)	0.0024 (4)	0.0108 (4)	0.0015 (4)
O2	0.0227 (5)	0.0175 (5)	0.0170 (5)	0.0062 (4)	0.0086 (4)	−0.0006 (4)
O3	0.0182 (5)	0.0164 (5)	0.0125 (5)	−0.0004 (4)	0.0051 (4)	−0.0035 (4)
O4	0.0221 (5)	0.0243 (6)	0.0133 (5)	−0.0105 (4)	0.0094 (4)	−0.0049 (4)
S1	0.0129 (2)	0.0119 (2)	0.0090 (2)	0	0.00474 (17)	0
O1W	0.0228 (6)	0.0202 (5)	0.0187 (6)	0.0065 (4)	0.0117 (5)	0.0044 (4)

### Geometric parameters (Å, °)

**Table:**

C1—O1	1.2144 (18)	N3—H3	0.86
C1—O2	1.3098 (17)	N4—H4A	0.86
C1—C2	1.4905 (19)	N4—H4B	0.86
C2—N2	1.3046 (18)	O2—H2	0.82
C2—N3	1.3648 (18)	O3—S1	1.4674 (10)
C3—N4	1.3236 (19)	O4—S1	1.4941 (10)
C3—N1	1.3420 (18)	S1—O3^i^	1.4674 (10)
C3—N3	1.3480 (18)	S1—O4^i^	1.4941 (10)
N1—N2	1.3774 (17)	O1W—H1W	0.89 (2)
N1—H1	0.86	O1W—H2W	0.83 (2)
			
O1—C1—O2	127.86 (13)	C3—N3—H3	126.9
O1—C1—C2	120.61 (13)	C2—N3—H3	126.9
O2—C1—C2	111.50 (12)	C3—N4—H4A	120
N2—C2—N3	112.37 (12)	C3—N4—H4B	120
N2—C2—C1	125.24 (13)	H4A—N4—H4B	120
N3—C2—C1	122.38 (12)	C1—O2—H2	109.5
N4—C3—N1	127.67 (13)	O3—S1—O3^i^	112.76 (9)
N4—C3—N3	125.69 (13)	O3—S1—O4	109.00 (6)
N1—C3—N3	106.63 (12)	O3^i^—S1—O4	108.98 (6)
C3—N1—N2	110.85 (11)	O3—S1—O4^i^	108.98 (6)
C3—N1—H1	124.6	O3^i^—S1—O4^i^	109.00 (6)
N2—N1—H1	124.6	O4—S1—O4^i^	108.01 (9)
C2—N2—N1	103.94 (11)	H1W—O1W—H2W	106.3 (19)
C3—N3—C2	106.20 (11)		
			
O1—C1—C2—N2	−179.54 (13)	C1—C2—N2—N1	−179.34 (12)
O2—C1—C2—N2	2.06 (19)	C3—N1—N2—C2	−0.42 (14)
O1—C1—C2—N3	1.6 (2)	N4—C3—N3—C2	177.99 (13)
O2—C1—C2—N3	−176.82 (12)	N1—C3—N3—C2	−1.19 (14)
N4—C3—N1—N2	−178.13 (13)	N2—C2—N3—C3	1.00 (16)
N3—C3—N1—N2	1.03 (15)	C1—C2—N3—C3	−179.99 (12)
N3—C2—N2—N1	−0.37 (15)		

Symmetry codes: (i) −*x*+1, *y*, −*z*+3/2.

### Hydrogen-bond geometry (Å, °)

**Table:**

*D*—H···*A*	*D*—H	H···*A*	*D*···*A*	*D*—H···*A*
O1W—H2W···N2	0.83 (2)	2.16 (2)	2.9774 (16)	169 (2)
O1W—H1W···O1^ii^	0.89 (2)	1.93 (2)	2.7941 (17)	163 (2)
O2—H2···O1W^iii^	0.82	1.74	2.5403 (16)	165
N1—H1···O4^iv^	0.86	1.94	2.7107 (16)	149
N3—H3···O4^v^	0.86	1.79	2.6436 (15)	171
N4—H4A···O3^vi^	0.86	2.17	2.8452 (17)	136
N4—H4B···O3^vii^	0.86	2.08	2.9255 (16)	168

Symmetry codes: (ii) *x*, −*y*+1, *z*−1/2; (iii) −*x*+1/2, −*y*+3/2, −*z*+1; (iv) −*x*+1, −*y*+1, −*z*+1; (v) −*x*+1, *y*−1, −*z*+3/2; (vi) −*x*+1, −*y*, −*z*+1; (vii) *x*, *y*−1, *z*.
